# The association between lipid accumulation product and osteoporosis in American adults: analysis from NHANES dataset

**DOI:** 10.3389/fmed.2025.1513375

**Published:** 2025-03-19

**Authors:** Huawen Pan, Xiao Long, Ping Wu, Yongchun Xiao, Huanran Liao, Li Wan, Jianxian Luo, Zhisheng Ji

**Affiliations:** ^1^Department of Orthopedics, First Affiliated Hospital of Jinan University, Guangzhou, China; ^2^Department of Spine Surgery, Maoming People's Hospital, Maoming, China; ^3^Department of Orthopedics, First Affiliated Hospital of Guangdong Pharmaceutical University, Guangzhou, China

**Keywords:** osteoporosis, lipid accumulation product, triglyceride, cross-sectional study, NHANES

## Abstract

**Background:**

The Lipid Accumulation Product (LAP), a novel indicator of fat accumulation, reflects the distribution and metabolic status of body fat. This study aims to evaluate the relationship between adult Americans’ prevalence of osteoporosis and LAP.

**Methods:**

This study used data from the NHANES cycles 2007–2010, 2013–2014, and 2017–2018, including 4,200 adults aged 50 and above. LAP was calculated using waist circumference and triglyceride levels, whereas osteoporosis was identified using information from dual-energy X-ray absorptiometry (DXA) assessments of bone mineral density (BMD). Restricted cubic spline (RCS) analysis was evaluated the relationship between LAP and osteoporosis. Additionally, subgroup analyses were conducted to assess the impact of demographic characteristics and health status on the relationship between LAP and osteoporosis.

**Results:**

LAP and osteoporosis were shown to be significantly inversely correlated in the study. In the unadjusted model, the prevalence of osteoporosis and Log LAP showed a significant negative connection (OR = 0.62, 95% CI = 0.52–0.74). Osteoporosis prevalence decreased by 45% in the fully adjusted model for every unit rise in Log LAP (OR = 0.54, 95% CI = 0.44–0.66). RCS analysis revealed a nonlinear association between LAP and osteoporosis prevalence (*P*-non-linear = 0.0025), showing an L-shaped negative correlation. Subgroup studies showed that, regardless of age, sex, ethnicity, or health condition, there was a constant negative connection between LAP and osteoporosis.

**Conclusion:**

According to this study, there is a substantial negative relationship between adult prevalence of osteoporosis in America and LAP. LAP is an easy-to-use and practical indication that may be very helpful in osteoporosis prevention and early detection.

## 1 Introduction

Decreased bone density and the breakdown of bone microarchitecture are the hallmarks of osteoporosis, a systemic skeletal disease that raises the risk of fractures dramatically and decreases bone mass (1–3). As the world’s population ages more rapidly, osteoporosis is becoming a serious global public health problem (4, 5). Osteoporosis is particularly prevalent among postmenopausal elderly women, primarily due to estrogen deficiency (6, 7). The World Health Organization (WHO) estimates that 200 million men and women worldwide suffer from osteoporosis (8). In addition to severely lowering patients’ quality of life, osteoporosis places a financial strain on healthcare systems (9, 10). The incidence of osteoporosis and fractures can be decreased by early identification and management.

Currently, dual-energy X-ray absorptiometry (DXA) is the gold standard for diagnosing osteoporosis. However, its application in large-scale screening is limited due to its high cost, equipment requirements, and low accessibility (11). Additionally, traditional risk assessment tools, such as the Fracture Risk Assessment Tool (FRAX), can predict fracture risk but remain controversial regarding their accuracy and applicability (12). Therefore, identifying an economical, convenient, and widely applicable biomarker for early screening and risk assessment of osteoporosis holds significant clinical value.

The Lipid Accumulation Product (LAP) is a novel indicator of fat accumulation that integrates waist circumference and serum triglyceride levels, providing a more accurate reflection of an individual’s fat distribution and metabolic status (13–15). BMI and body fat percentage cannot differentiate the effects of different fat types on bone health (16). Previous studies have demonstrated a stronger association between visceral fat and bone metabolism. Moreover, LAP has been validated as a predictive marker for metabolic syndrome-related diseases, including diabetes, non-alcoholic fatty liver disease, and hypertension (17–20), suggesting its potential value in osteoporosis screening. However, systematic studies on the relationship between LAP and osteoporosis remain limited, particularly with regard to epidemiological evidence in the U.S. adult population.

This study utilizes data from the National Health and Nutrition Examination Survey (NHANES) to systematically assess the relationship between LAP and osteoporosis prevalence, aiming to explore the feasibility of LAP as a potential biomarker for early osteoporosis screening (21–23). Because hormones and cytokines secreted by visceral adipose tissue, such as leptin, may promote bone formation by stimulating osteoblasts (24), and a moderate increase in fat mass may enhance skeletal loading, thereby stimulating bone remodeling and increasing bone density (25). We hypothesize that LAP is significantly associated with bone mineral density (BMD), with higher LAP levels potentially correlating with a lower risk of osteoporosis. By further investigating this association, we aim to provide new theoretical insights for osteoporosis screening and prevention, as well as support future clinical practice and public health policies.

## 2 Methods

### 2.1 Survey description

The National Health and Nutrition Examination Survey (NHANES), a cross-sectional survey, uses a complex, stratified, multistage sampling procedure to assess the overall health and nutritional status of the American population. Each participant provided signed, informed consent, and the trial was authorized by the Institutional Review Board.

### 2.2 Study population

Data from four NHANES cycles—2007–2010, 2013–2014, and 2017–2018—were used in this analysis. The absence of femoral bone density measurements led to the exclusion of data from the 2011–2012 and 2015–2016 cycles. The inclusion criteria were: (1) participants aged over 50; (2) participants with complete femoral bone density measurements; and (3) participants with complete waist circumference and triglyceride data.

### 2.3 Calculation of LAP

Using waist circumference (WC) and triglyceride (TG) values, the LAP index is computed using the formula presented in earlier research. This is the formula for calculation: The formula for calculating LAP is [WC (cm) - 65] × TG (mmol/l) for males and [WC (cm) - 58] for women. × TG in mol/l.

### 2.4 Definition of osteoporosis

Using mobile examination facilities, NHANES performed DXA scans on the proximal femur to collect data on bone mineral density (BMD) in the trochanter, femoral neck, whole hip, and intertrochanteric areas. In accordance with WHO recommendations, a T-score of less than −2.5 standard deviations in total hip BMD, femoral neck BMD, trochanter BMD, or intertrochanteric BMD indicates osteoporosis. The reference group is made up of white, non-Hispanic women between the ages of 20 and 29. The following formula is used to determine the T-score: T-Score is calculated as standard deviation / (individual BMD - mean normal BMD).

### 2.5 Covariates

This study considers demographic characteristics, lifestyle, health status, and laboratory tests as covariates. The poverty index ratio (PIR), age, sex, race, and educational attainment are examples of demographic characteristics. The PIR is categorized as <1, 1 to <3, and ≥ 3 based on the results. Lifestyle factors include smoking and physical activity. A smoker is defined as someone who has smoked more than 100 cigarettes in their lifetime. The following method is used to calculate physical activity in metabolic equivalent of task (MET) minutes per week based on data from the Global Physical Activity Questionnaire: MET (minutes/week) is calculated as follows: MET value x weekly frequency × session time. A MET value less than 600 min/week is defined as inactive. Other health conditions are determined based on physician diagnosis records or self-reports, including diabetes, hypertension, and chronic kidney disease. Laboratory tests include blood uric acid, blood urea nitrogen, blood creatinine, alanine aminotransferase, aspartate aminotransferase, blood calcium, and blood phosphorus concentrations.

### 2.6 Statistical analysis

Analysis was based on NHANES data from 2007 to 2018, excluding the 2011–2012 and 2015–2016 cycles with missing femoral bone density data. Baseline data were displayed based on whether osteoporosis was present or absent using descriptive analysis. Categorical variables were displayed as percentages, while continuous variables were displayed as means and standard deviations. Supplementary Figure 5 revealed that the LAP data presented a left-skewed distribution. LAP was logarithmically transformed to correct the data skew and standardize the data. The corrected data are shown in Supplementary Figure 6. After controlling for confounders, the association between LAP and osteoporosis was examined using logistic regression. LAP was converted into a four-category variable to explore trends with osteoporosis at different levels and enhance result robustness. Saturation effect analysis was used to determine the critical point and restricted cubic spline (RCS) analysis was used to assess the dose–response relationship between LAP and osteoporosis. The possible effects of age, sex, race, smoking, physical activity, diabetes, chronic renal disease, and hypertension on the correlation between Log LAP and osteoporosis were examined using subgroup analysis. More study was done to look into the relationship between Log LAP and BMD in different femur locations using linear regression and RCS analysis in order to increase result robustness and consistency. *p* < 0.05 was used as the significance criterion, and all analyses were conducted using R software (version 4.2.3).

## 3 Results

### 3.1 Characteristics of study population

**Figure 1:**
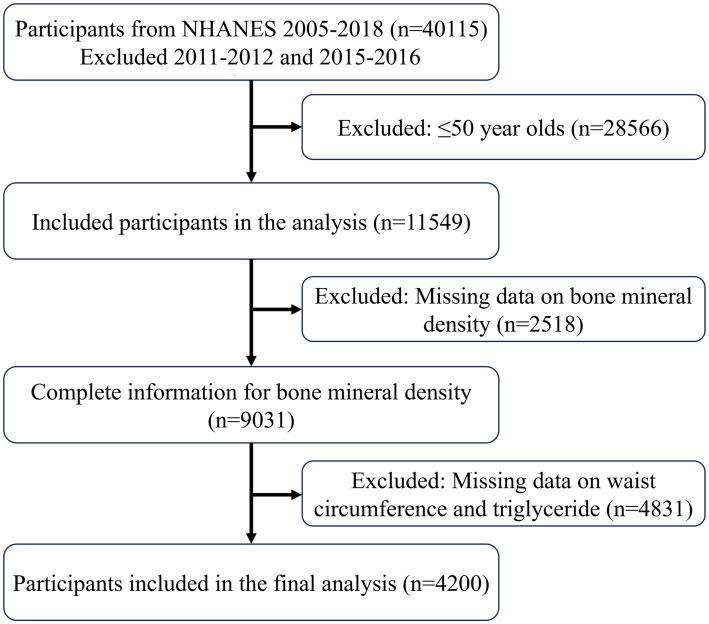
Include participants in the process.

Data extracted from the NHANES database followed the inclusion process as shown in Figure 1, ultimately including 4,200 participants, with 3,826 classified as non-osteoporotic and 374 as osteoporotic. Supplementary Table 1 presents weighted baseline characteristics based on the osteoporosis categorization, whereas Table 1 presents participant characteristics. In contrast to the non-osteoporotic group, the osteoporotic individuals tended to be non-Hispanic white, more females than men, and older overall. Individuals with osteoporosis tended to lead more sedentary lives and had elevated blood phosphorus and blood urea nitrogen levels. It is noteworthy that osteoporotic participants had lower LAP levels, suggesting LAP may be a protective factor against osteoporosis.

**Table 1:** Baseline characteristics of the study population.

Characteristic	Overall	Non-osteoporosis	Osteoporosis	*p*-value
*n*	4,200	3,826	374	
Age (%)				<0.001
<65	2,315 (55.1)	2,213 (57.8)	102 (27.3)	
>65	1885 (44.9)	1,613 (42.2)	272 (72.7)	
Sex (%)				<0.001
Female	2068 (49.2)	1809 (47.3)	259 (69.3)	
Male	2,132 (50.8)	2017 (52.7)	115 (30.7)	
Race (%)				<0.001
Mexican American	554 (13.2)	515 (13.5)	39 (10.4)	
Non-Hispanic black	806 (19.2)	777 (20.3)	29 (7.8)	
Non-Hispanic white	2008 (47.8)	1774 (46.4)	234 (62.6)	
Others	832 (19.8)	760 (19.9)	72 (19.3)	
Education level (%)				<0.001
Under high school	1,186 (28.2)	1,056 (27.6)	130 (34.8)	
High school or equivalent	986 (23.5)	882 (23.1)	104 (27.8)	
Above high school	2021 (48.1)	1884 (49.2)	137 (36.6)	
PIR (%)				<0.001
<1	613 (16.3)	543 (15.9)	70 (20.5)	
1–3	1,670 (44.5)	1,490 (43.6)	180 (52.8)	
>3	1,474 (39.2)	1,383 (40.5)	91 (26.7)	
Activity status (%)				<0.001
Active	1766 (42.0)	1,656 (43.3)	110 (29.4)	
Inactive	2,434 (58.0)	2,170 (56.7)	264 (70.6)	
Smoke (%)				0.144
No	2094 (49.9)	1896 (49.6)	198 (52.9)	
Yes	2,103 (50.1)	1928 (50.4)	175 (46.8)	
Hypertension (%)				0.74
No	1919 (45.7)	1751 (45.8)	168 (44.9)	
Yes	2,276 (54.2)	2070 (54.1)	206 (55.1)	
CKD (%)				<0.001
No	4,007 (95.4)	3,668 (95.9)	339 (90.6)	
Yes	184 (4.4)	150 (3.9)	34 (9.1)	
Diabate (%)				0.146
No	3,236 (77.0)	2,934 (76.7)	302 (80.7)	
Yes	808 (19.2)	745 (19.5)	63 (16.8)	
Total femur BMD [mean (SD)] (gm/cm2)	0.92 (0.16)	0.95 (0.15)	0.66 (0.09)	<0.001
Femoral neck BMD [mean (SD)] (gm/cm2)	0.76 (0.14)	0.78 (0.13)	0.53 (0.05)	<0.001
Trochanter BMD [mean (SD)] (gm/cm2)	0.70 (0.14)	0.72 (0.13)	0.50 (0.08)	<0.001
Intertrochanter BMD [mean (SD)] (gm/cm2)	1.10 (0.19)	1.13 (0.17)	0.79 (0.12)	<0.001
BUN [mean (SD)] (mmol/L)	5.50 (2.31)	5.44 (2.24)	6.09 (2.86)	<0.001
ALT [mean (SD)] (IU/L)	23.79 (15.30)	24.10 (15.16)	20.59 (16.40)	<0.001
AST [mean (SD)] (U/L)	25.36 (13.18)	25.42 (13.37)	24.77 (11.12)	0.365
SCR [mean (SD)] (umol/L)	0.95 (0.47)	0.95 (0.46)	0.97 (0.51)	0.273
SUA [mean (SD)] (mg/dL)	5.70 (1.44)	5.73 (1.44)	5.35 (1.46)	<0.001
Calcium [mean (SD)] (mmol/L)	2.35 (0.09)	2.35 (0.09)	2.34 (0.10)	0.122
Phosphorus [mean (SD)] (mmol/L)	1.17 (0.17)	1.17 (0.17)	1.21 (0.17)	<0.001
WC [mean (SD)] (cm)	100.29 (13.80)	101.05 (13.58)	92.51 (13.70)	<0.001
TG [mean (SD)] (mmol/L)	1.46 (1.10)	1.47 (1.14)	1.31 (0.66)	0.007
LAP [mean (SD)]	58.38 (52.46)	59.73 (53.80)	44.55 (32.93)	<0.001

Mean (SD) for continuous variables, % for categorical variables.

ALT, alanine aminotransferase; AST, aspartate aminotransferase; BUN, blood urea nitrogen; CKD, chronic kidney disease; LAP, lipid accumulation product; PIR, poverty income ratio; SCR, serum creatinine; SUA, serum uric acid; TG, triglyceride; WC, waist circumference.

### 3.2 Association between LAP and prevalence of osteoporosis

To correct the left-skewed data of LAP, we performed a logarithmic transformation (Log LAP) on LAP. The results of a logistic regression study that looked at the relationship between Osteoporosis Prevalence and Log LAP are shown in Table 2. Log LAP showed a significant negative correlation (OR = 0.62, 95% CI = 0.52–0.74) with the frequency of osteoporosis in the unadjusted model (Model 1). Once covariates were corrected for step-by-step, the completely adjusted model (Model 3) showed that for every unit increase in Log LAP, the prevalence of osteoporosis decreased by 45% (OR = 0.54, 95% CI = 0.44–0.66). Further analysis after converting LAP into a categorical variable revealed that as LAP levels increased, the prevalence of osteoporosis significantly decreased (P-trend <0.001). Even after adjusting for every other variable, there was still a significant negative correlation between the highest quartile of LAP levels and the prevalence of osteoporosis (OR = 0.33, 95% CI = 0.19–0.55).

**Table 2:** The relationship between log LAP and osteoporosis.

		Model 1 OR (95%CI) *P*-value	Model 2 OR (95%CI) *P*-value	Model 3 OR (95%CI) *P*-value
Osteoporosis	log LAP	0.62 (0.52, 0.74) <0.001	0.57 (0.47, 0.70) <0.001	0.54 (0.44, 0.67) <0.001
	Q1	[Reference]	[Reference]	[Reference]
	Q2	0.68 (0.49, 0.94) 0.020	0.65 (0.46, 0.93) 0.018	0.65 (0.44, 0.94) 0.025
	Q3	0.55 (0.37, 0.80) 0.002	0.46 (0.31, 0.69) <0.001	0.45 (0.28, 0.73) 0.002
	Q4	0.41 (0.26, 0.64) <0.001	0.39 (0.25, 0.59) <0.001	0.33 (0.20, 0.55) <0.001
	P for trend	<0.001	<0.001	<0.001

CI, confidence interval; LAP, lipid accumulation product; OR, odds ratio; Q, quartiles.

Model 1: No covariates adjusted.

Model 2: Adjusted for age, sex, and race.

Model 3: Adjusted for age, sex, race, educational level, PIR, calcium, phosphorus, smoke, hypertension, CKD, diabetes, SCR, BUN, SUA, AST, ALT.

### 3.3 Nonlinear relationship and saturation effect analysis

**Figure 2:**
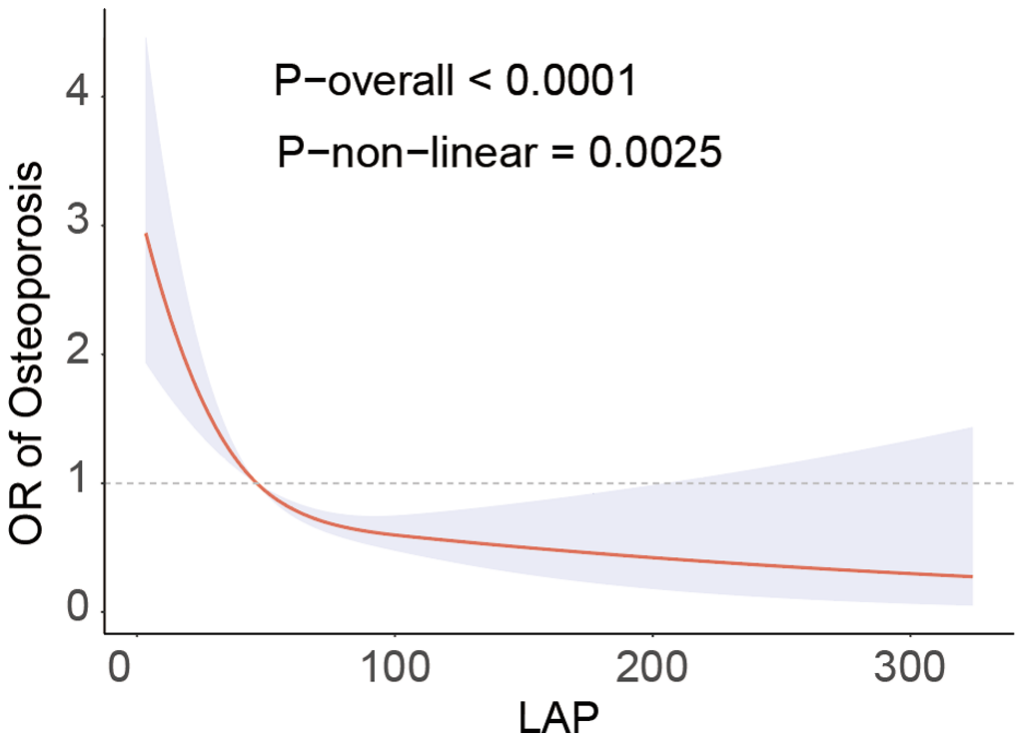
RCS analysis fitted the relationship between LAP and osteoporosis. Adjusted for age, sex, race, educational level, PIR, calcium, phosphorus, smoke, hypertension, CKD, diabetes, SCR, BUN, SUA, AST, ALT.

The RCS analysis revealed a nonlinear association between LAP and the prevalence of osteoporosis (*P*-non-linear = 0.0025), presenting an L-shaped negative correlation (Figure 2). Through threshold effect analysis, we identified a turning point of LAP = 29 in the osteoporosis population. Segmental logistic regression analysis (Table 3) showed that when LAP <29, an increase in LAP was significantly associated with a reduced prevalence of osteoporosis (OR = 0.95, 95% CI = 0.93–0.97). However, when LAP >29, the effect of increasing LAP on osteoporosis prevalence gradually weakened (OR = 0.99, 95% CI = 0.99–1.00). These findings suggest a negative association with a saturation threshold between LAP and osteoporosis.

**Table 3:** Analysis of the LAP saturation effect and osteoporosis.

	LAP	OR (95%CI) *P*-value
Osteoporosis	Standard linear model	0.99 (0.98, 0.99) <0.001
	LAP <29	0.95 (0.93, 0.97) <0.001
	LAP >29	0.99 (0.99, 1.00) <0.001
	Log-likelihood ratio test	<0.001

LAP, lipid accumulation product; OR, odds ratio; CI, confidence interval.

Adjusted for age, sex, race, educational level, PIR, calcium, phosphorus, smoke, hypertension, CKD, diabetes, SCR, BUN, SUA, AST, ALT.

### 3.4 Subgroup analysis

**Figure 3:**
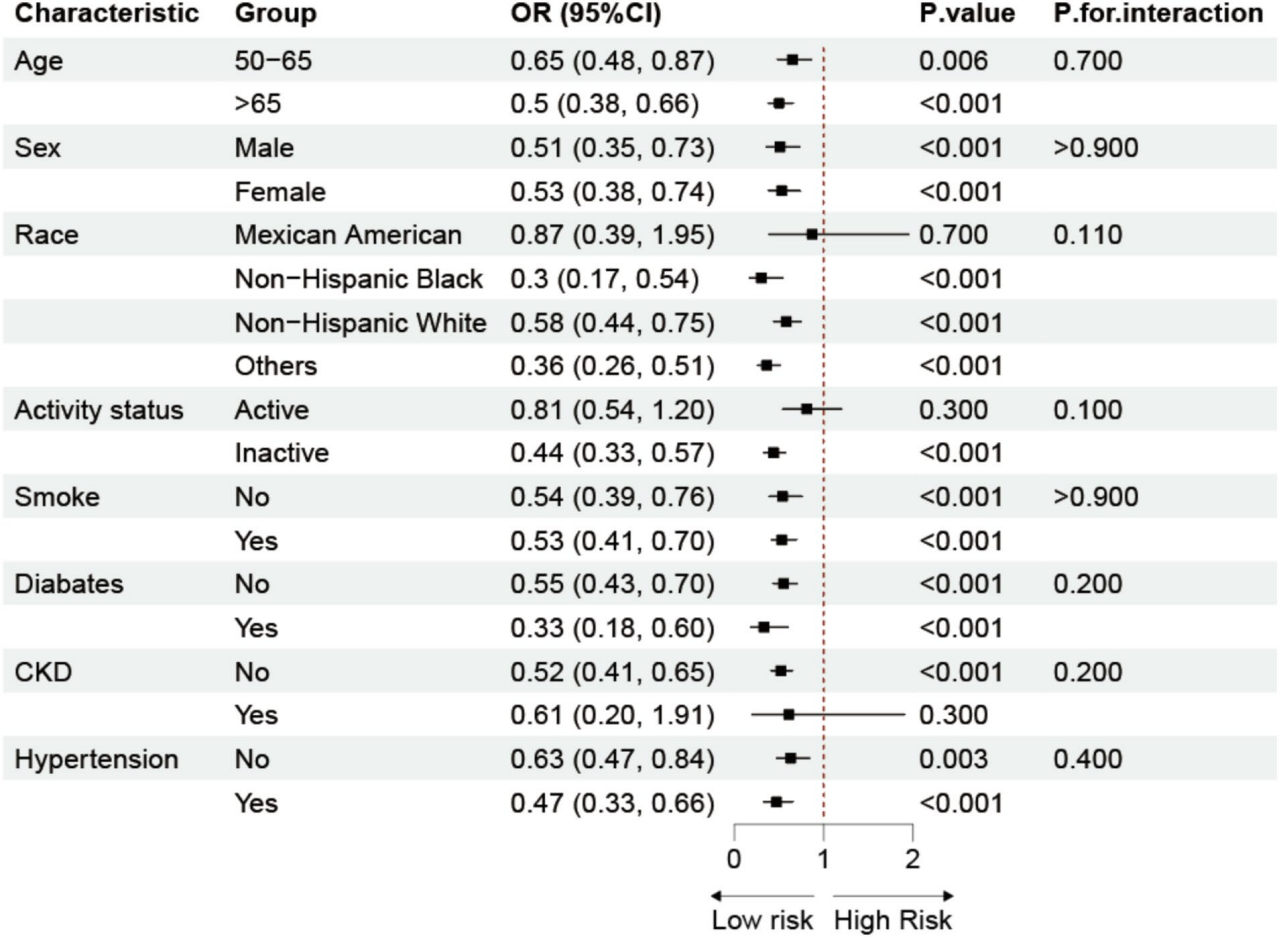
Subgroup analysis of the association between LAP and osteoporosis. Adjusted for age, sex, race, educational level, PIR, calcium, phosphorus, smoke, hypertension, CKD, diabetes, SCR, BUN, SUA, AST, ALT.

We performed a subgroup analysis using Model 3 (Figure 3) in conjunction with stratification variables such age, gender, race, physical activity, smoking, diabetes, chronic kidney disease, and hypertension to look into any possible associations between Log LAP and osteoporosis. The findings demonstrated that there was a persistent negative correlation between the prevalence of osteoporosis and Log LAP. Additionally, the interaction tests did not yield statistically significant results, suggesting that Log LAP may operate as a stand-alone protective factor against osteoporosis.

### 3.5 Additivity analysis

To verify the robustness and consistency of our findings, we conducted additional analyses. Supplementary Table 2 shows that Log LAP is positively and consistently associated with BMD in different regions of the femur, indicating that Log LAP is a favorable factor for BMD. Supplementary Figures 1–4 illustrate the RCS analyses of LAP and BMD in different regions of the femur, demonstrating nonlinear relationships and threshold saturation effects, thus supporting the robustness and consistency of our study results.

## 4 Discussion

This study used data from the NHANES database to examine the relationship between the prevalence of osteoporosis and LAP. The findings demonstrated a strong inverse relationship between osteoporosis and LAP. An increase in LAP considerably decreased the incidence of osteoporosis in the lower range of LAP; however, this protective impact progressively diminished when LAP above a certain threshold. These findings suggest that moderate fat distribution may positively impact bone health by increasing mechanical loading on bones and promoting the secretion of bone-forming factors.

LAP is an indicator of fat accumulation that combines waist circumference and triglyceride levels, effectively reflecting an individual’s visceral fat level (26, 27). Compared to the traditional BMI, LAP has higher accuracy and sensitivity in assessing metabolic health and cardiovascular disease risk (28, 29). In line with certain other research findings, we discovered a strong negative connection between LAP and osteoporosis in our study. Similarly, in a study of the Visceral Adiposity Index (VAI) in adults over 20 years of age, those with high VAI scores also had high total femur BMD, suggesting that those with higher levels of visceral adiposity had a lower risk of fracture (30). Another study suggests that moderate weight gain protects bone density by increasing the mechanical load on bone through adipose tissue and by promoting bone formation through the secretion of several hormones and cytokines (31). In addition, the accumulation of visceral fat may have a positive effect on bone health by promoting the secretion of hormones such as leptin, which inhibits osteoclast activity (32, 33).

On the other hand, excessive fat buildup may be detrimental to bone health (34). High fat impaired bone mass and some trabecular microstructures in older mice in an experimental study of older mice (35). In another mechanistic study, the obesity-related adipose tissue secretes hormones and inflammatory substances such TNF-*α* and interleukin-6 (IL-6), which can raise the risk of osteoporosis by accelerating bone resorption and preventing the production of new bone (36–38). Excess fat may lead to adverse effects such as chronic inflammation, oxidative stress, and insulin resistance, ultimately accelerating bone loss (39). Particularly at higher LAP levels, these negative effects might outweigh the positive ones, leading to an increased risk of osteoporosis. Therefore, the nonlinear relationship between LAP and osteoporosis observed in this study suggests that the protective effect of fat accumulation on bone health diminishes or even disappears when fat accumulation reaches a certain level.

We carried out several more studies to confirm the consistency and robustness of our findings. First, we used restricted cubic spline (RCS) analysis to look at the dose–response relationship between LAP and osteoporosis (40, 41). The results showed that the risk of osteoporosis was significantly reduced for each unit increase in LAP when LAP <29 (OR = 0.95, 95% CI = 0.93–0.97, *p* < 0.001); however, this protective effect gradually diminished when LAP >29. Additionally, we conducted a subgroup analysis to explore the potential influence of variables such as age, sex, race, physical activity, smoking, diabetes, chronic kidney disease, and hypertension on the relationship between LAP and osteoporosis. The results indicated a consistent negative correlation between LAP and osteoporosis across different populations, suggesting that LAP may serve as an independent protective factor. This phenomenon may be attributed to the fact that LAP more directly reflects the metabolic status of visceral fat.

In assessing the connection between LAP and osteoporosis risk in adult Americans, this study identified LAP as a stand-alone protective factor against osteoporosis. The large sample size enhances the accuracy of our conclusions. Additionally, LAP outperforms traditional BMI in assessing individual fat distribution. Moreover, we adjusted for various confounding variables based on demographic characteristics and chronic diseases to minimize confounding bias, ensuring the broad applicability of the findings and enhancing both the internal and external validity of the study. In order to further understand the connection between LAP and osteoporosis in various groups, we lastly performed stratified subgroup analyses. These results highlight the necessity for more targeted osteoporosis preventive measures. There are many restrictions on this study. First of all, it is hard to establish a direct correlation between LAP and osteoporosis due to the cross-sectional nature of the study. Subsequent long-term investigations are required to confirm these results and investigate the possible utility of LAP in osteoporosis care and prevention. Secondly, this study used DXA to measure BMD and defined osteoporosis based on WHO criteria. Although DXA is considered the “gold standard” for osteoporosis diagnosis, it only assesses bone mineral density and does not evaluate bone quality or microarchitecture. Since fracture risk is also influenced by factors such as trabecular structure and cortical thickness, relying solely on BMD may underestimate the fracture risk in some patients. Due to the limitations of DXA, this study could not further explore the impact of LAP on bone microarchitecture. Future studies may integrate HR-pQCT or bone turnover markers to optimize osteoporosis risk assessment. Finally, as a novel body composition indicator, LAP requires further research to support its clinical application. This study found that LAP may be an independent protective factor against osteoporosis, as moderate fat distribution benefits bone health, while obesity may have adverse effects. LAP, being simple and practical, holds promise for osteoporosis prevention and early detection. Future studies should validate its applicability across different populations and its value in osteoporosis risk assessment.

## 5 Conclusion

According to this study, there is a substantial negative relationship between adult prevalence of osteoporosis in America and LAP. LAP is an easy-to-use and practical indication that may be very helpful in osteoporosis prevention and early detection.

## Data Availability

Publicly available datasets were analyzed in this study. This data can be found at: National Center for Health Statistics: www.cdc.gov/nchs/nhanes/.
