# Supplementary material for: The association between lipid accumulation product and osteoporosis in American adults: analysis from NHANES dataset
Source: Front Med (Lausanne). 2025 Mar 19;12:1513375. doi: 10.3389/fmed.2025.1513375 (PMC11961649; doi:10.3389/fmed.2025.1513375)
Supplement: Supplementary file 1 [file Data_Sheet_1.docx]

**Supplementary Table 1: Weighted baseline characteristics of the study population**

| Characteristic | Overall | Non-Osteoporosis | Osteoporosis | P-value |
| --- | --- | --- | --- | --- |
| n | 36712452.9 | 33811742.9 | 2900709.9 |  |
| Age (%) |  |  |  | <0.001 |
| <65 | 22536629.6 (61.4) | 21625929.8 (64.0) | 910699.8 (31.4) |  |
| >65 | 14175823.3 (38.6) | 12185813.1 (36.0) | 1990010.1 (68.6) |  |
| Sex (%) |  |  |  | <0.001 |
| Female | 19148912.6 (52.2) | 17038984.2 (50.4) | 2109928.4 (72.7) |  |
| Male | 17563540.3 (47.8) | 16772758.7 (49.6) | 790781.6 (27.3) |  |
| Race (%) |  |  |  | 0.001 |
| Mexican American | 1847339.9 (5.0) | 1731615.5 (5.1) | 115724.4 (4.0) |  |
| Non-Hispanic black | 3313229.8 (9.0) | 3202574.7 (9.5) | 110655.2 (3.8) |  |
| Non-Hispanic white | 27503100.0 (74.9) | 25167069.6 (74.4) | 2336030.4 (80.5) |  |
| Others | 4048783.2 (11.0) | 3710483.1 (11.0) | 338300.0 (11.7) |  |
| Education level (%) |  |  |  | <0.001 |
| Under high school | 6252841.6 (17.0) | 5509813.4 (16.3) | 743028.1 (25.6) |  |
| High school or equivalent | 9303170.3 (25.3) | 8415494.2 (24.9) | 887676.1 (30.6) |  |
| Above high school | 21140361.2 (57.6) | 19876430.3 (58.8) | 1263930.9 (43.6) |  |
| PIR (%) |  |  |  | <0.001 |
| <1 | 3312297.3 (9.8) | 2856580.9 (9.2) | 455716.4 (16.6) |  |
| 1-3 | 12092687.8 (35.9) | 10748738.6 (34.8) | 1343949.2 (48.8) |  |
| >3 | 18241563.1 (54.2) | 17289438.1 (56.0) | 952125.0 (34.6) |  |
| Activity status (%) |  |  |  | <0.001 |
| Active | 17078620.7 (46.5) | 16095918.4 (47.6) | 982702.3 (33.9) |  |
| Inactive | 19633832.2 (53.5) | 17715824.6 (52.4) | 1918007.6 (66.1) |  |
| Smoke (%) |  |  |  | 0.001 |
| No | 18388962.5 (50.1) | 16952737.6 (50.1) | 1436224.9 (49.5) |  |
| Yes | 18303057.7 (49.9) | 16854618.2 (49.8) | 1448439.5 (49.9) |  |
| Hypertension (%) |  |  |  | 0.576 |
| No | 18283754.3 (49.8) | 16922343.8 (50.0) | 1361410.4 (46.9) |  |
| Yes | 18389201.1 (50.1) | 16849901.6 (49.8) | 1539299.5 (53.1) |  |
| Hypercholesterolemia (%) |  |  |  | 0.409 |
| No | 15832575.5 (45.5) | 14495914.0 (45.2) | 1336661.4 (48.8) |  |
| Yes | 18746051.6 (53.9) | 17372008.8 (54.2) | 1374042.9 (50.2) |  |
| CKD (%) |  |  |  | <0.001 |
| No | 35542657.7 (96.8) | 32876799.1 (97.2) | 2665858.6 (91.9) |  |
| Yes | 1121177.6 (3.1) | 889324.9 (2.6) | 231852.7 (8.0) |  |
| Diabate (%) |  |  |  | 0.136 |
| No | 29878318.6 (81.4) | 27414927.2 (81.1) | 2463391.4 (84.9) |  |
| Yes | 5497382.4 (15.0) | 5116602.6 (15.1) | 380779.8 (13.1) |  |
| Total femur BMD (mean (SD)) (gm/cm2) | 0.92 (0.16) | 0.94 (0.14) | 0.67 (0.09) | <0.001 |
| Femoral neck BMD (mean (SD)) (gm/cm2) | 0.76 (0.14) | 0.78 (0.13) | 0.53 (0.05) | <0.001 |
| Trochanter BMD (mean (SD)) (gm/cm2) | 0.70 (0.13) | 0.71 (0.12) | 0.50 (0.08) | <0.001 |
| Intertrochanter BMD (mean (SD)) (gm/cm2) | 1.09 (0.19) | 1.12 (0.17) | 0.80 (0.12) | <0.001 |
| BUN (mean (SD)) (mmol/L) | 5.50 (2.09) | 5.46 (2.01) | 6.00 (2.79) | <0.001 |
| ALT (mean (SD)) (IU/L) | 24.12 (15.43) | 24.42 (15.44) | 20.56 (14.86) | <0.001 |
| AST (mean (SD)) (U/L) | 25.28 (13.22) | 25.34 (13.33) | 24.60 (11.78) | 0.337 |
| SCR (mean (SD)) (μmol/L) | 0.92 (0.37) | 0.92 (0.36) | 0.96 (0.49) | 0.053 |
| SUA (mean (SD)) (mg/dL) | 5.63 (1.42) | 5.66 (1.40) | 5.32 (1.57) | 0.005 |
| Calcium (mean (SD)) (mmol/L) | 2.35 (0.09) | 2.35 (0.09) | 2.34 (0.09) | 0.331 |
| Phosphorus (mean (SD)) (mmol/L) | 1.18 (0.18) | 1.18 (0.18) | 1.20 (0.17) | 0.023 |
| WC (mean (SD)) (cm) | 100.40 (14.19) | 101.10 (14.03) | 92.25 (13.45) | <0.001 |
| TG (mean (SD)) (mmol/L) | 1.43 (1.07) | 1.44 (1.09) | 1.30 (0.67) | 0.010 |
| LAP (mean (SD)) | 58.30 (53.07) | 59.46 (54.20) | 44.79 (34.79) | <0.001 |

Mean (SD) for continuous variables, % for categorical variables.

ALT: Alanine Aminotransferase; AST: Aspartate Aminotransferase; BUN: Blood Urea Nitrogen; CKD: Chronic kidney disease; LAP: lipid accumulation product; PIR, Poverty Income Ratio; SCR: Serum Creatinine; SUA: Serum Uric acid; TG: Triglyceride; WC: Waist Circumference.

**Supplementary Table 2 Additional analysis between BRI and BMD.**

|  |  | Model 1  OR (95%CI) P-value | Model 2  OR (95%CI) P-value | Model 3  OR (95%CI) P-value |
| --- | --- | --- | --- | --- |
| Total femur BMD | Log LAP | 0.06 (0.05, 0.07) <0.001 | 0.06 (0.06, 0.07) <0.001 | 0.06 (0.05, 0.06) <0.001 |
|  | Q1 | [Reference] | [Reference] | [Reference] |
|  | Q2 | 0.06 (0.04, 0.07) <0.001 | 0.05 (0.04, 0.07) <0.001 | 0.05 (0.03, 0.06) <0.001 |
|  | Q3 | 0.07 (0.06, 0.09) <0.001 | 0.08 (0.06, 0.09) <0.001 | 0.06 (0.05, 0.07) <0.001 |
|  | Q4 | 0.12 (0.10, 0.13) <0.001 | 0.12 (0.10, 0.13) <0.001 | 0.11 (0.09, 0.12) <0.001 |
|  | P for trend | <0.001 | <0.001 | <0.001 |
| Femoral neck BMD | Log LAP | 0.04 (0.03, 0.05) <0.001 | 0.04 (0.04, 0.05) <0.001 | 0.04 (0.03, 0.05) <0.001 |
|  | Q1 | [Reference] | [Reference] | [Reference] |
|  | Q2 | 0.03 (0.02, 0.05) <0.001 | 0.03 (0.02, 0.05) <0.001 | 0.03 (0.01, 0.04) <0.001 |
|  | Q3 | 0.04 (0.03, 0.06) <0.001 | 0.05 (0.04, 0.06) <0.001 | 0.04 (0.03, 0.05) <0.001 |
|  | Q4 | 0.08 (0.06, 0.09) <0.001 | 0.08 (0.07, 0.10) <0.001 | 0.07 (0.06, 0.09) <0.001 |
|  | P for trend | <0.001 | <0.001 | <0.001 |
| Trochanter BMD | Log LAP | 0.05 (0.04, 0.06) <0.001 | 0.05 (0.04, 0.05) <0.001 | 0.05 (0.04, 0.05) <0.001 |
|  | Q1 | [Reference] | [Reference] | [Reference] |
|  | Q2 | 0.05 (0.03, 0.06) <0.001 | 0.04 (0.03, 0.06) <0.001 | 0.04 (0.03, 0.06) <0.001 |
|  | Q3 | 0.06 (0.04, 0.07) <0.001 | 0.06 (0.05, 0.07) <0.001 | 0.05 (0.04, 0.06) <0.001 |
|  | Q4 | 0.09 (0.08, 0.11) <0.001 | 0.09 (0.08, 0.10) <0.001 | 0.09 (0.07, 0.10) <0.001 |
|  | P for trend | <0.001 | <0.001 | <0.001 |
| Intertrochanter BMD | Log LAP | 0.07 (0.06, 0.08) <0.001 | 0.07 (0.07, 0.08) <0.001 | 0.07 (0.06, 0.07) <0.001 |
|  | Q1 | [Reference] | [Reference] | [Reference] |
|  | Q2 | 0.07 (0.05, 0.09) <0.001 | 0.06 (0.04, 0.08) <0.001 | 0.05 (0.03, 0.07) <0.001 |
|  | Q3 | 0.09 (0.07, 0.11) <0.001 | 0.09 (0.07, 0.11) <0.001 | 0.07 (0.05, 0.09) <0.001 |
|  | Q4 | 0.14 (0.11, 0.16) <0.001 | 0.14 (0.12, 0.16) <0.001 | 0.12 (0.10, 0.14) <0.001 |
|  | P for trend | <0.001 | <0.001 | <0.001 |

CI: Confidence Interval; LAP: Lipid Accumulation Product; OR: Odds Ratio; Q: Quartiles

Model 1: No covariates adjusted; Model 2: Adjusted for Age, Sex, and Race; Model 3: Adjusted for age, sex, race, educational level, PIR, calcium, phosphorus, smoke, hypertension, CKD, diabetes, SCR, BUN, SUA, AST, ALT.


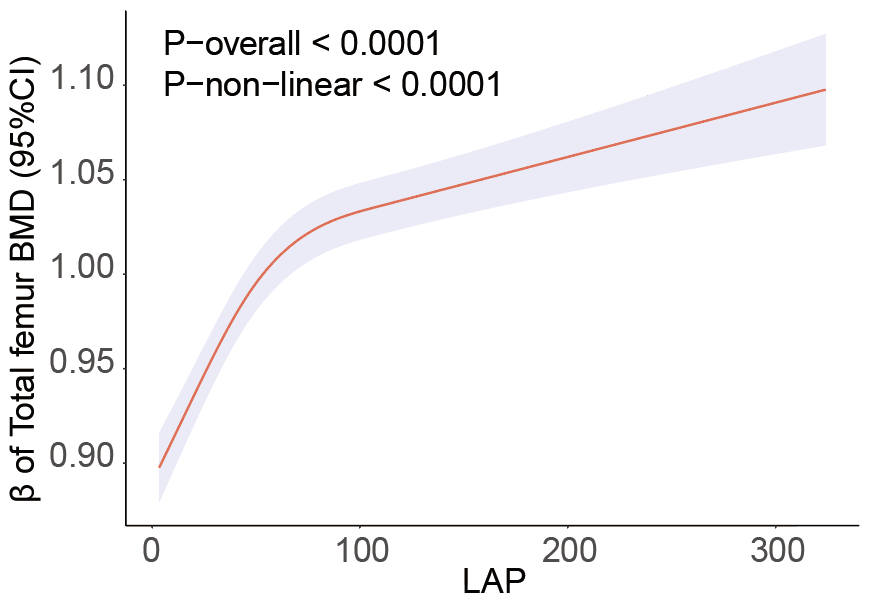


**Supplementary figure 1: RCS analysis fitted the relationship between LAP and Total femur BMD.**

Adjusted for age, sex, race, educational level, PIR, calcium, phosphorus, smoke, hypertension, CKD, diabetes, SCR, BUN, SUA, AST, ALT.


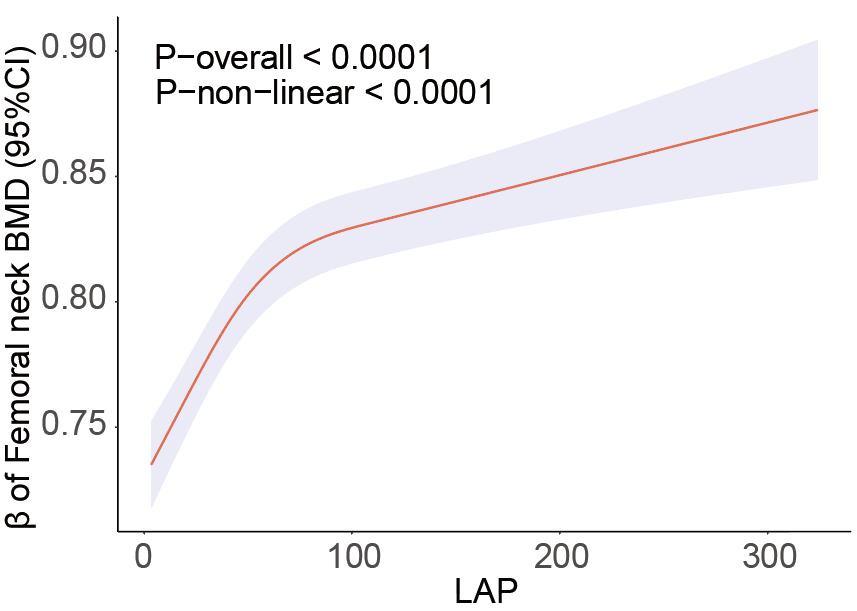


**Supplementary figure 2: RCS analysis fitted the relationship between LAP and Femoral neck BMD.**

Adjusted for age, sex, race, educational level, PIR, calcium, phosphorus, smoke, hypertension, CKD, diabetes, SCR, BUN, SUA, AST, ALT.


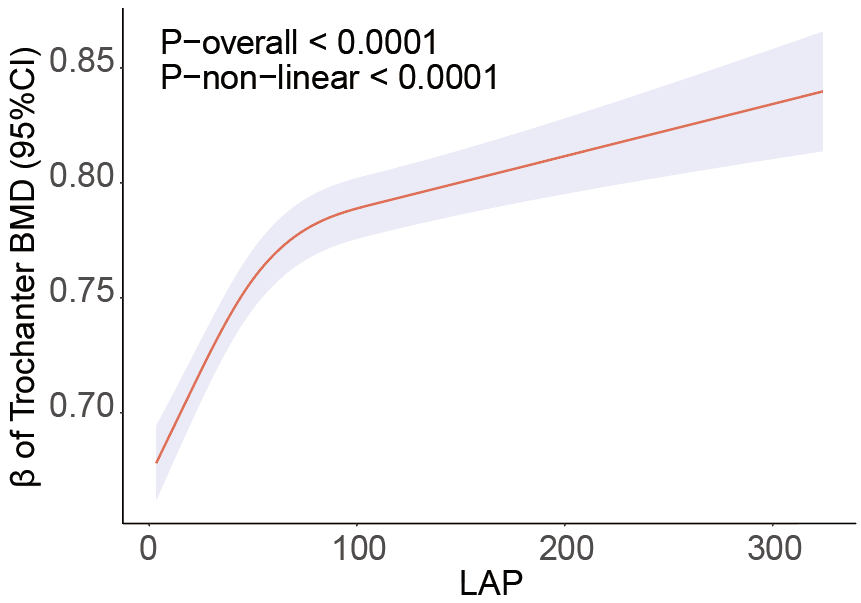
 **Supplementary figure 3: RCS analysis fitted the relationship between LAP and Trochanter BMD.**

Adjusted for age, sex, race, educational level, PIR, calcium, phosphorus, smoke, hypertension, CKD, diabetes, SCR, BUN, SUA, AST, ALT.


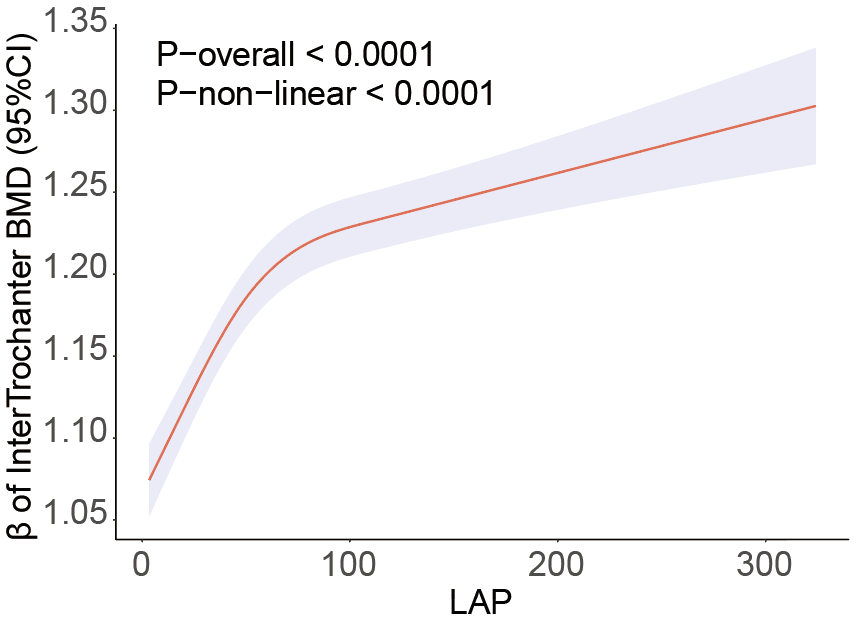
 **Supplementary figure 4: RCS analysis fitted the relationship between LAP and Intertrochanter BMD.**

Adjusted for age, sex, race, educational level, PIR, calcium, phosphorus, smoke, hypertension, CKD, diabetes, SCR, BUN, SUA, AST, ALT.


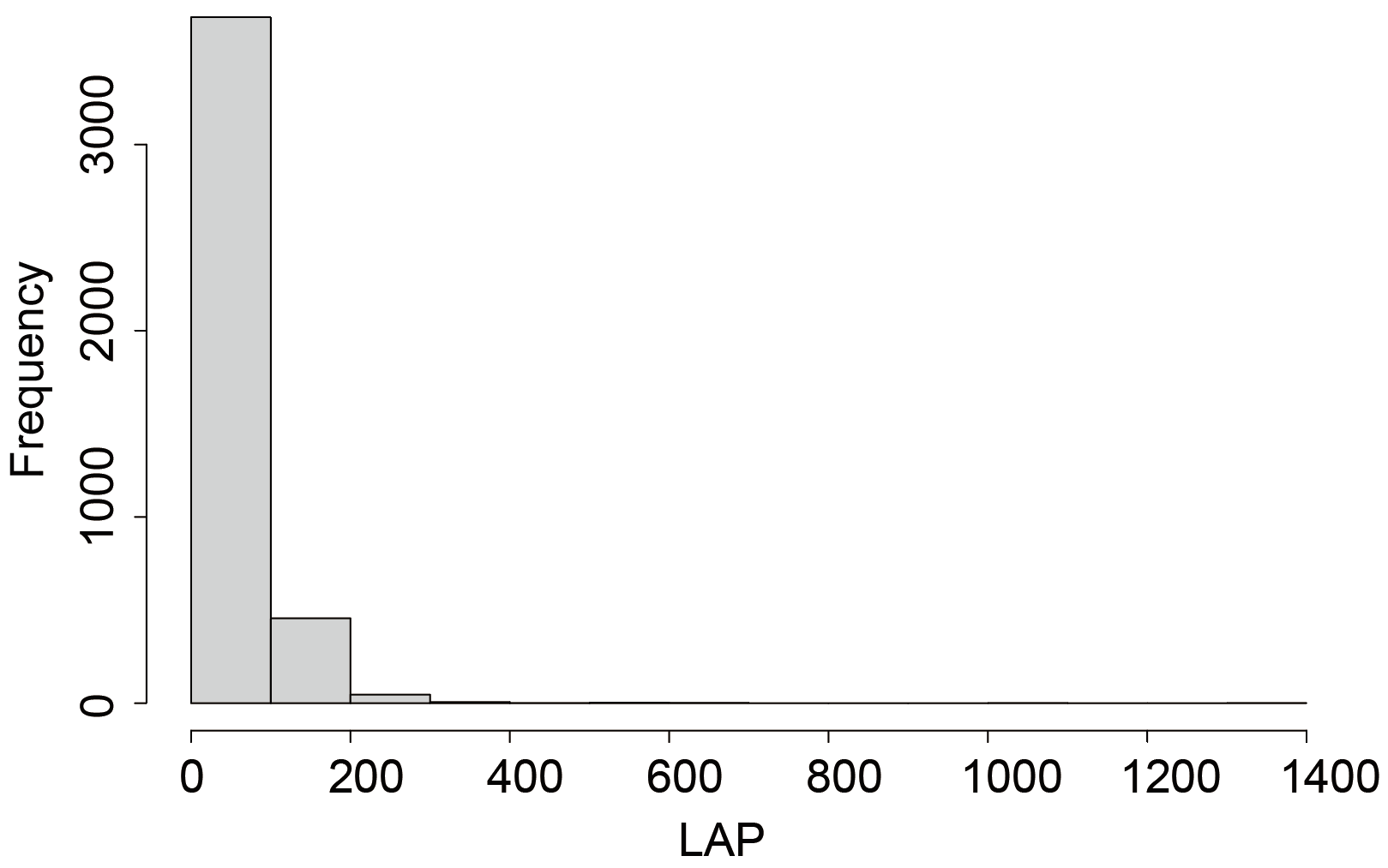


**Supplementary figure 5: Histogram of LAP**


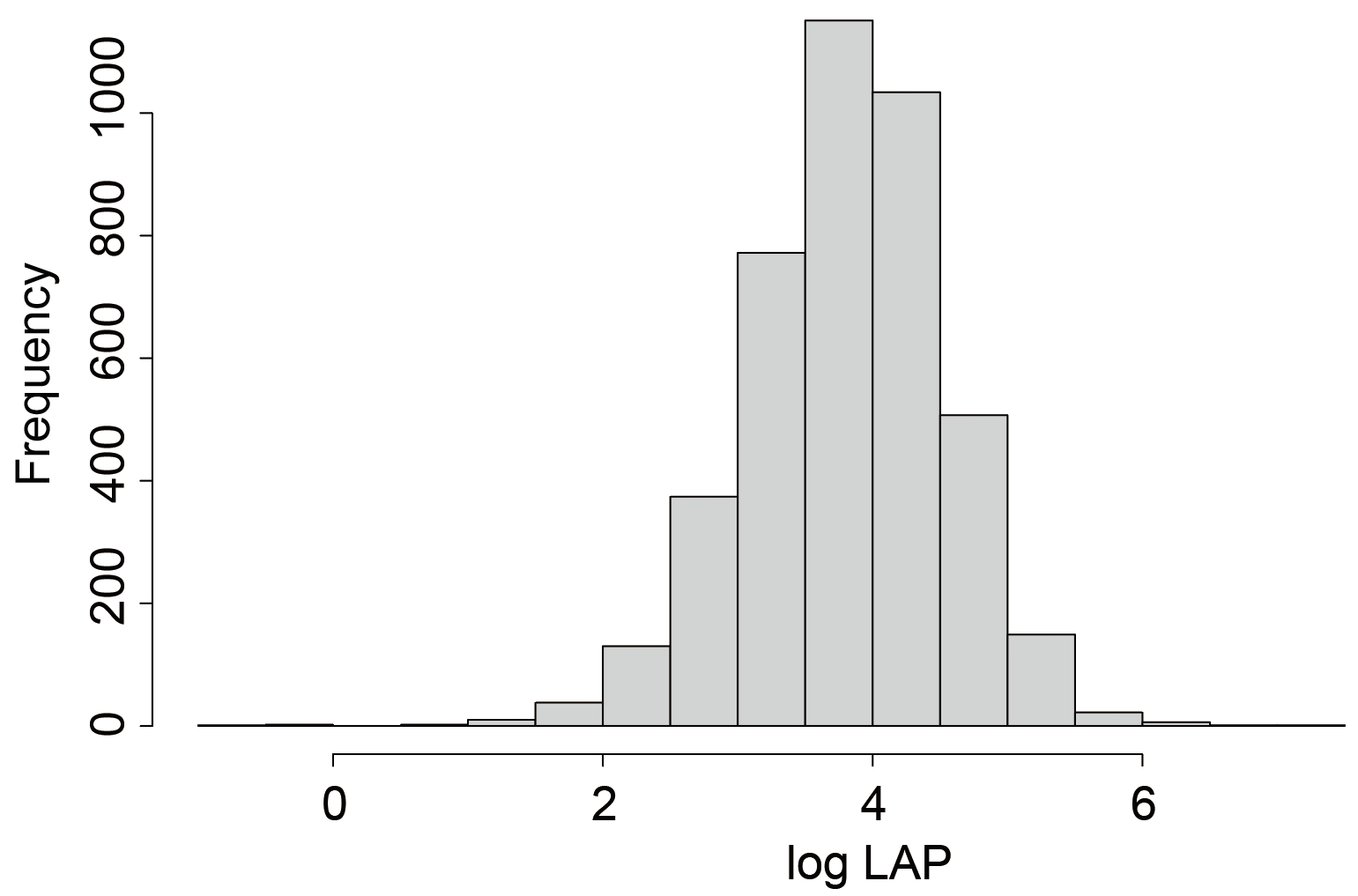


**Supplementary figure 6: Histogram of log LAP**
